# Author Correction: Proton irradiation-decelerated intergranular corrosion of Ni-Cr alloys in molten salt

**DOI:** 10.1038/s41467-024-47902-4

**Published:** 2024-04-25

**Authors:** Weiyue Zhou, Yang Yang, Guiqiu Zheng, Kevin B. Woller, Peter W. Stahle, Andrew M. Minor, Michael P. Short

**Affiliations:** 1 Department of Nuclear Science and Engineering, Massachusetts Institute of Technology, Cambridge, MA USA; 2 National Center for Electron Microscopy, Molecular Foundry, Lawrence Berkeley National Laboratory, Berkeley, CA USA; 3 Nuclear Reactor Laboratory, Massachusetts Institute of Technology, Cambridge, MA USA; 4 Department of Materials Science and Engineering, University of California, Berkeley, CA USA

Correction to: *Nature Communications* 10.1038/s41467-020-17244-y, published online 9 July 2020

In the original version of this article, numbers for the beam current densities were incorrectly given as 1.5, 2.0, and 2.5 mA cm^−2^ in various locations, instead of the correct values 0.3, 0.4, and 0.5 mA cm^−2^. This was owing to a measurement error coming from the indirect correspondence between the Faraday cup and the beam profile monitor (BPM) on the accelerator as the proton beam traveling along the beamline resulted in mismatching between calculated values and real beam currents, which were obtained by ex-situ calibration.

The following changes have been made in the correct version.

The eighth sentence of the Results, and the figure legend of Fig. 2j state ‘0.3, 0.4, 0.5 mA cm^−2^’ in place of ‘1.5, 2.0, 2.5 mA cm^−2^’.

The figure legend of Fig. 1j–l states ‘0.5, 0.4, 0.3 mA cm^−2^’ in place of ‘2.5, 2.0, 1.5 mA cm^−2^’.

Figure 1e states ‘0.5 mA cm^−2^’ in place of ‘2.5 mA cm^−2^’, Fig. 1h states ‘0.4 mA cm^−2^’ in place of ‘2.0 mA cm^−2^’, and Fig. 1k states ‘0.3 mA cm^−2^’ in place of ‘1.5 mA cm^−2^’.

Figure 2c states ‘0.5 mA cm^−2^’ in place of ‘2.5 mA cm^−2^’, Fig. 2d states ‘0.4 mA cm^−2^’ in place of ‘2.0 mA cm^−2^’, and Fig. 2e states ‘0.3 mA cm^−2^’ in place of ‘1.5 mA cm^−2^’.

Figure 2j and k states ‘0.3 mA cm^−2^’, ‘0.4 mA cm^−2^’, ‘0.5 mA cm^−2^’ in place of ‘1.5 mA cm^−2^, ‘2.0 mA cm^−2^’, and ‘2.5 mA cm^−2^’, respectively.

The figure legend of Fig. 3b states ‘0.4 mA cm^−2^’ in place of ‘2.0 mA cm^−2^’.

The figure legend of Supplementary Fig. 1 states ‘0.5 mA cm^−2^’ in place of ‘2.5 mA cm^−2^’.

This has been corrected in the PDF and HTML versions of the Article.

### Supplementary information

Updated Supplementary Information

