# Supplementary material for: Proton irradiation-decelerated intergranular corrosion of Ni-Cr alloys in molten salt
Source: Nat Commun. 2020 Jul 9;11:3430. doi: 10.1038/s41467-020-17244-y (PMC7347530; doi:10.1038/s41467-020-17244-y)
Supplement: Supplementary file 1 — Supplementary Information [file 41467_2020_17244_MOESM1_ESM.pdf]

**Supplementary Information**

# Proton Irradiation-Decelerated Intergranular Corrosion of Ni-Cr Alloys in Molten Salt

Zhou et al.

# Supplementary Discussion

## Mechanistic and quantitative differences between radiation-induced segregation (RIS) and a molten salt corrosion system under irradiation

Materials under irradiation can undergo elemental segregation across grain boundaries (GBs) and other features, known as radiation-induced segregation (RIS) [1]. RIS is often explained by Inverse Kirkendall (IK) theory [2], where different elements preferentially couple to either the interstitial flux or vacancy flux. The similarly directional fluxes of vacancies and interstitials towards GBs drive the elemental segregation resulting in RIS. In a Ni-Cr binary alloy system undergoing RIS, Ni is predicted and observed to be enriched, and Cr depleted [3, 4]. This would have the effect of driving Ni towards GBs, making them more inert to molten salt corrosion. The qualitative argument therefore exists that RIS could be responsible for the observed Ni and Cr movement in our system. However, a quantitative examination of the rate of RIS shows that another mechanism must be primarily responsible.

Quantitatively speaking, one of the best datasets and models of RIS in alloys with concentrations similar to ours is that of Allen et al. [4, 5], who modeled RIS in Ni-18Cr-9Fe at temperatures between 200-600°C to doses between 0.1-3.0 Displacements Per Atom (DPA). Data from the model in Fig. 4 of Ref. [4] were extracted using the WebPlotDigitizer [6] for the case of Cr depletion in Ni-18Cr-9Fe at 400°C, to investigate the extent of Cr depletion solely due to RIS. Comparing their Auger electron spectroscopy (AES) validated model results to ours at an experimental proton dose of 0.01 DPA, 0.45% Cr depletion would be expected based on the rate of RIS in these materials at 400°C (see Fig. 4 of [4]). This should be far lower at higher temperatures, as shown in Fig. 5 of the same reference, as it is well known that RIS subsides at higher temperatures due to chemical back-diffusion and high recombination rates of defects [7]. In fact at 650°C RIS should be nearly non-existent in Ni-18Cr-9Fe [4], and it should be similarly insignificant in our Ni-20Cr at 650°C. What we observe in Supplementary Figure 2, roughly a 7-8% depletion in measured Cr concentration, is therefore unable to be explained by RIS alone in Ni-based alloys of similar Cr concentration. Data extracted from Fig. 4 of [4] can be found in the data repository for this manuscript [8].

Since RIS could not have generated the elemental distribution profile which we observed in Supplementary Figure 2, corrosion driven elemental fluxes must therefore be responsible. As described in the main text, both Cr/Ni elemental fluxes and a corrosion-injected vacancy flux exist in a system undergoing corrosion via selective removal of Cr. The former would still exist in a closed system undergoing RIS, while the latter would only exist in an open system undergoing corrosion. In our case, selective removal of Cr by the molten salt injects vacancies into the GBs, creating copious free volume. Cr and Ni then move via this injected vacancy flux, which is away from GBs. This is fundamentally different from an RIS process, as it happens in both irradiated and unirradiated systems. When adding irradiation, the preferential coupling of Ni to the radiation-induced interstitial flux and Cr to the radiation-induced vacancy flux probably also occurs in the molten salt corrosion system under irradiation. It is unlikely that this preferential defect coupling is dominant in our system, given the duration of the experiment and the low level of damage. Diffusion, however, is greatly enhanced in proportion to defect creation, both by radiation and by vacancy injection from the corrosion process. The presence of radiation defects therefore accelerates the transport of both Cr and Ni towards the GBs primarily in response to Cr depletion and free volume creation due to corrosion. This transport still occurs without irradiation, though the addition of a significant interstitial flux hastens the process in rough proportion to the total, elevated defect concentration.

Thus the preferential coupling between defect fluxes and atom fluxes can still occur in our experiments. However, the coupling of both Ni and Cr atoms to defect fluxes — against vacancy flux and follow interstitial flux — likely dominates over the preferential coupling. Therefore, radiation enhanced diffusion, coupled to the corrosion induced diffusion process, is recognized as the mechanism behind the deceleration phenomenon.

# Supplementary Figures

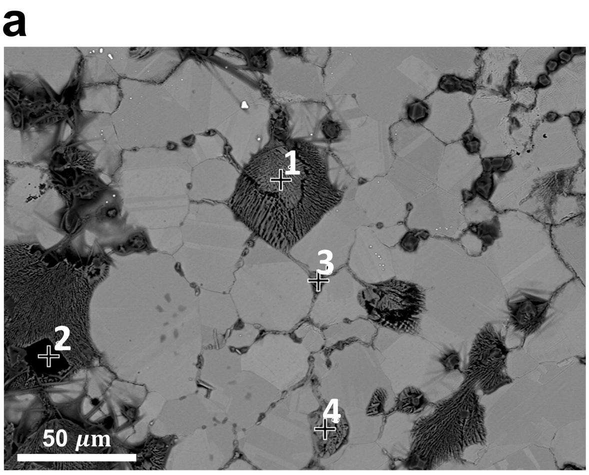


**b**

| Spots | Detected Major Elements |
|---|---|
| Spot 1 | F, K, Na, Eu |
| Spot 2 | F |
| Spot 3 | F, K, Na, Cr |
| Spot 4 | F, K, Eu |

Supplementary Figure 1. **Elemental identification of the salt components from SEM/EDX measurements on the beam facing side.**
**(a)** SEM image of the unirradiated zone of the beam-facing side of the Ni-20Cr foil after 4 hours at 650°C under 0.5 μA cm$^{-2}$, showing spots for the EDX measurements. Scale bar: 50 μm **(b)** Summary of the EDX measurements, showing detected major elements.

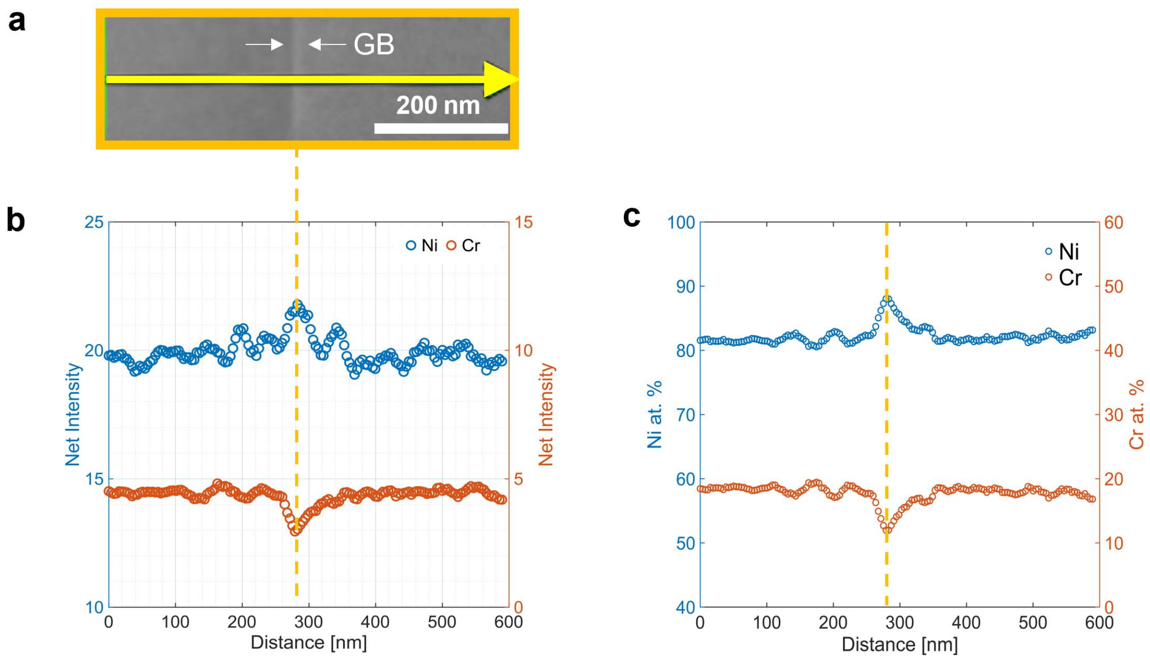


Supplementary Figure 2. **Elemental distribution results from TEM/EDX measurements across a corroded grain boundary in the irradiated zone.**
**(a)** STEM image of a characteristic grain boundary, as indicated by the white arrow. The yellow line marks the EDX linescan. Scale bar: 200 nm. **(b)** EDX net intensity profile for the line shown in (a). **(c)** EDX atomic percentage profile for the line shown in (a).

## Supplementary References


[1] M. Nastar and F. Soisson, Comprehensive Nuclear Materials **1**, 471 (2012).
[2] H. Wiedersich, P. R. Okamoto, and N. Q. Lam, J. Nucl. Mater. **83**, 98 (1979).
[3] C. M. Barr, L. Barnard, J. E. Nathaniel, K. Hattar, K. A. Unocic, I. Szlurfarska, D. Morgan, and M. L. Taheri, J. Mater. Res. **30**, 1290 (2015).
[4] T. R. Allen and G. S. Was, Acta Mater. **46**, 3679 (1998).
[5] T. R. Allen, J. T. Busby, G. S. Was, and E. A. Kenik, J. Nucl. Mater. **255**, 44 (1998).
[6] A. Rohatgi, "Webplotdigitizer," (2011), version 4.2 (April 2019), Accessible at https://automeris.io/WebPlotDigitizer.
[7] G. S. Was, *Fundamentals of radiation materials science: metals and alloys* (Springer, 2016).
[8] W. Y. Zhou, Y. Yang, G. Q. Zheng, P. W. Stahle, K. B. Woller, A. M. Minor, and M. P. Short, (2019), GitHub Repository for This Manuscript, accessible at https://github.com/shortlab/2019-Radiation-Decelerated-Corrosion, DOI: 10.5281/zenodo.3579494.
